# Primitive Erythropoiesis in the Mouse is Independent of DOT1L Methyltransferase Activity

**DOI:** 10.3389/fcell.2021.813503

**Published:** 2022-01-17

**Authors:** Carrie A. Malcom, Anamika Ratri, Joanna Piasecka-Srader, Shaon Borosha, V. Praveen Chakravarthi, Nehemiah S. Alvarez, Jay L. Vivian, Timothy A. Fields, M.A. Karim Rumi, Patrick E. Fields

**Affiliations:** Department of Pathology and Laboratory medicine, University of Kansas Medical Center, Kansas City, KS, United States

**Keywords:** DOT1L methyltransferase, Dot1l methyltransferase mutant mouse, erythroid progenitor, erythroid differentiation, early blood development

## Abstract

DOT1-like (DOT1L) histone methyltransferase is essential for mammalian erythropoiesis. Loss of DOT1L in knockout (*Dot1l-*KO) mouse embryos resulted in lethal anemia at midgestational age. The only recognized molecular function of DOT1L is its methylation of histone H3 lysine 79 (H3K79). We generated a *Dot1l* methyltransferase mutant (*Dot1l-MM*) mouse model to determine the role of DOT1L methyltransferase activity in early embryonic hematopoiesis. *Dot1l-MM* embryos failed to survive beyond embryonic day 13.5 (E13.5), similarly to *Dot1l-*KO mice. However, when examined at E10.5, *Dot1l-MM* embryos did not exhibit overt anemia like the *Dot1l-KO*. Vascularity and the presence of red blood cells in the *Dot1l-MM* yolk sacs as well as in the AGM region of *Dot1l-MM* embryos appeared to be similar to that of wildtype. In *ex vivo* cultures of yolk sac cells, *Dot1l-MM* primitive erythroblasts formed colonies comparable to those of the wildtype. Although *ex vivo* cultures of *Dot1l-MM* definitive erythroblasts formed relatively smaller colonies, inhibition of DOT1L methyltransferase activity *in vivo* by administration of EPZ-5676 minimally affected the erythropoiesis. Our results indicate that early embryonic erythropoiesis in mammals requires a DOT1L function that is independent of its intrinsic methyltransferase activity.

## Introduction

DOT1-like (DOT1L) histone H3 lysine methyltransferase is essential for a number of biological processes during embryonic development, including early hematopoiesis (Feng et al., 2010; Cattaneo et al., 2016; Pursani et al., 2018; Sutter et al., 2021). We detected that DOT1L deficiency results in lethal anemia in *Dot1l* knockout (*Dot1l-KO*) mouse embryos starting at embryonic day 11.5 (E11.5) and they failed to thrive beyond E13.5 (Feng et al., 2010). *Dot1l-KO* erythroid-myeloid progenitors failed to develop normally, exhibiting slowed cell-cycle progression *in vitro* associated with downregulation of GATA2, a transcription factor essential for erythropoiesis, and upregulation of PU.1, another factor that inhibits erythropoiesis (Feng et al., 2010). However, the precise molecular mechanisms underlying DOT1L regulation of early hematopoiesis remain largely unknown (Malcom et al., 2020).

Expression of *Dot1/Dot1L* is conserved across species (Feng et al., 2002; van Leeuwen et al., 2002; Shanower et al., 2005; Janzen et al., 2006; Steger et al., 2008). A number of cellular processes including transcriptional elongation (Steger et al., 2008), cell-cycle progression (Barry et al., 2009; Kim et al., 2014; Yang et al., 2019), and repair of DNA damage (FitzGerald et al., 2011; Wakeman et al., 2012; Kari et al., 2019) can be regulated by DOT1L. However, the exact mechanisms of DOT1L regulation of these cellular processes remain unclear. The only accepted molecular function that has been demonstrated for DOT1L is the histone H3 lysine 79 (H3K79) methylation by its S-adenosyl-l-methionine (SAM)-dependent, methyltransferase domain (Singer et al., 1998; Ng et al., 2002; Min et al., 2003; Sawada et al., 2004). Histone methylation is important for permissive and repressive chromatin conformation, which can have profound effects on the regulation of gene expression (Jambhekar et al., 2019). DOT1L is the only known methyltransferase in eukaryotic cells responsible for the mono-, di- and tri-methylation of H3K79 (Feng et al., 2002). These histone H3 modifications are strongly associated with actively transcribed chromatin regions (Steger et al., 2008). However, DOT1L has also been associated with repression of gene transcription (Wong et al., 2015). Nevertheless, it remains unclear whether the methyltransferase activity or the H3K79 methylation is linked to transcriptional elongation, cell cycle progression, or DNA damage repair. It also remains undetermined if the methyltransferase activity or H3K79 methylation is required for the development of the various specialized embryonic tissues, such as blood (Feng et al., 2010; Cattaneo et al., 2016; Pursani et al., 2018; Sutter et al., 2021).

DOT1L is quite a large protein (∼1540aa) that possesses multiple potential domains for interacting with many epigenetic and transcriptional regulators (Chen et al., 2015; Castelli et al., 2018). In addition to the methyltransferase activity, such domains of DOT1L may also be linked to its diverse potential of cellular functions. In this study, we sought to determine if the methyltransferase activity of DOT1L that is responsible for H3K79 methylation, regulates early embryonic hematopoiesis. Using the CRISPR/Cas9 system, we generated a mouse line possessing a single amino acid substitution from asparagine to alanine at amino acid position 241(Asp241Ala). The mutation lies within the histone catalytic domain of mouse DOT1L methyltransferase and eliminates H3K79 methylation in mutant cells (Min et al., 2003). *Dot1l* methyltransferase mutant (*Dot1l-*MM) mice exhibited embryonic lethality similar to that of *Dot1l-KO* mice. However, unlike the *Dot1l-*KO (Feng et al., 2010), *Dot1l-*MM yolk sacs and embryos did not exhibit any significant deficiency in early embryonic blood formation.

## Materials and Methods

### Generation of *Dot1l-*MM Mouse Embryonic Stem Cells

**FIGURE 1:**
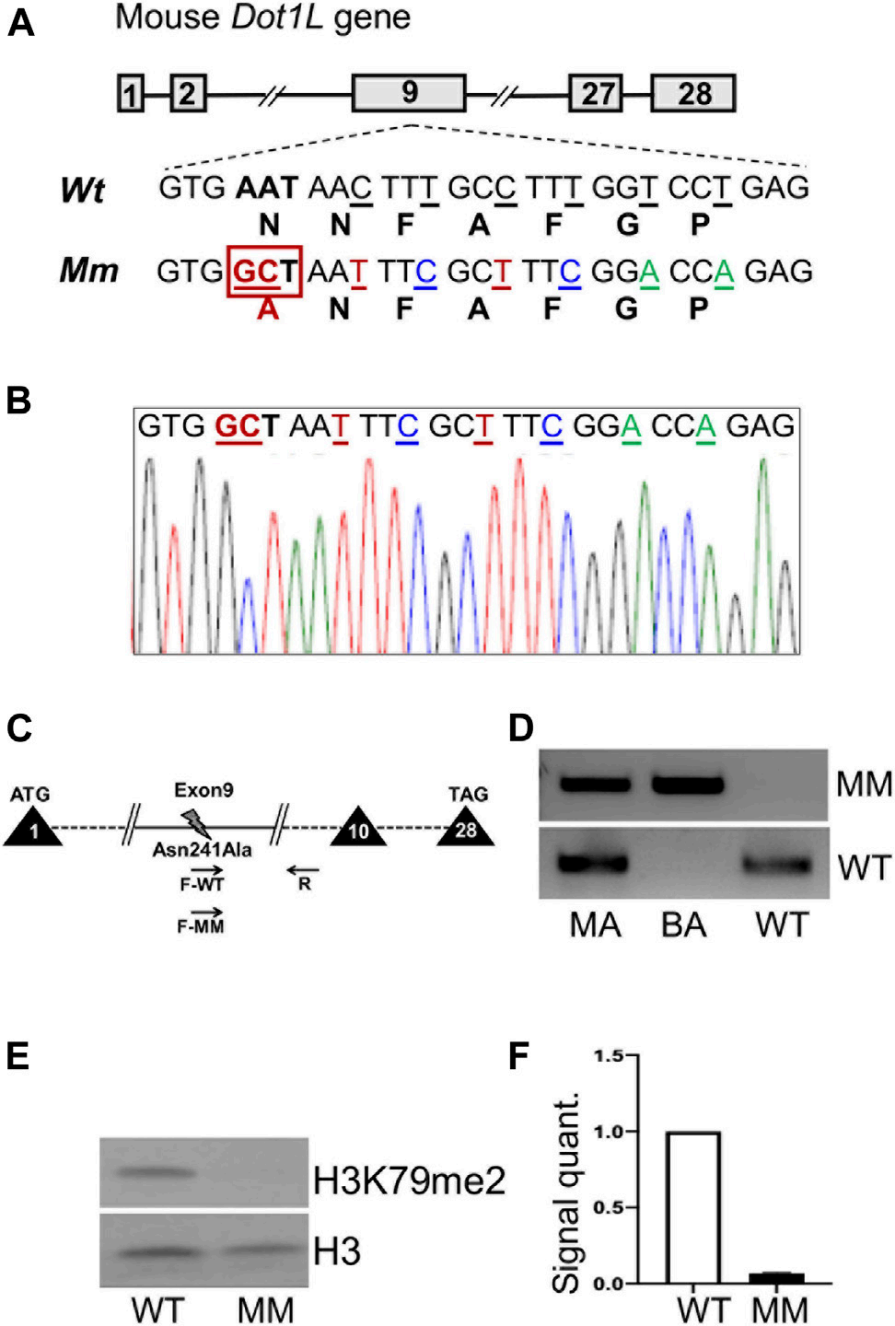
Generation of Dot1l-MM mouse embryonic stem cells (mESC). Mouse embryonic stem cell line, E14 was targeted via CRISPR/Cas9 to create a point mutation (Asp241Ala) in the coding sequence of the Dot1l gene **(A,B)**. The Methyl mutant (MM) allele contained an additional 6 silent nucleotide changes (underlined) to prevent targeting by the gRNA while generating the biallelic mutation **(A,B)**. Monoallelic and biallelic methyl mutant clones of mESC were identified by allele specific PCR **(C,D)**. Western blot analyses showed the absence of histone H3, lysine 79 di-methylation (H3K79me2) in biallelic, Dot1l-MM E14 cells **(E,F)**. Data presented as mean ± SEM, n ≥ 3, and * indicates p ≤ 0.05.

*Dot1l*-MM mouse embryonic stem (mES) cells were generated by electroporation of E14 TG2a (E14) cells (129/Ola) with targeted guide RNA (gRNA), Cas9 enzyme and a homologous recombination template DNA carrying the point mutation in exon nine to substitute the coding sequence of asparagine 241 to alanine (Asn241Ala) (Figures 1A,B). *Dot1l-MM* mES cell clones were identified by allele-specific PCR of genomic DNA (Figure 1C,D) and the mutant DNA sequence was verified by Sanger sequencing (Figure 1B). A biallelic mutant mES cell clone was evaluated for H3K79 di-methylation (Figures 1E,F). Another monoallelic mutant mES line was selected for microinjection into blastocysts to generate the *Dot1l-MM* mouse.

### Generation of *Dot1l-MM* Mice

The E14 mES cell clone carrying a single *Dot1l-MM* allele was microinjected into wildtype C57BL/6J mouse (Jackson Laboratories) blastocysts by the Transgenic Institutional Facility at KUMC to generate the *Dot1l-MM* chimeric mice. Chimeric mutant founders were identified by agouti (129/Ola) spots in C57BL/6J pups. *Dot1l-MM* heterozygous F1 mice were generated by breeding the chimeras with C57BL/6J mice. Stocks of *Dot1l-MM* heterozygous mice were maintained by backcrossing to 129 stocks (Charles River). All animal procedures were approved by the University of Kansas Medical Center Animal Care and Use Committee.

### Assessment of Embryonic Lethality and Formation of Red Blood Cells

Heterozygous *Dot1l-MM* male and female mice were setup for timed mating. Pregnant females were euthanized during mid-gestation starting from E10.5 through E13.5. Conceptuses were collected and dissected under stereomicroscopic examination to remove decidua. Cleaned conceptuses were numbered individually and photographed to visualize vascularization in yolk sacs and presence of red blood cells in yolk sacs. Then yolk sacs were opened to isolate the embryos and photographed to demonstrate the presence of red blood cells. Genomic DNA was extracted from the embryos and *Dot1l-MM* genotyping was performed by allele-specific PCR (Figures 1C,D). Genotyping results were used to determine the Mendelian ratio and to assess the viability.

### Analysis of Primitive Erythropoiesis in Yolk Sac

Primitive erythropoiesis was measured by performing colony-forming assays as described previously (Palis et al., 1999). Briefly, after timed mating of heterozygous *Dot1l-MM* male and females, E8.5 conceptuses were collected in IMDM. Embryos were used for genomic DNA extraction and genotyping. Yolk sacs were digested with 0.1% collagenase I (StemCell Technologies) in IMDM (Gibco-BRL) at 37°C for 30 min, neutralized with 10% ES grade FBS (Hyclone) and single cell suspensions were prepared by repeated pipetting. Yolk sac cells were washed with serum free IMDM and approximately equal numbers of homozygous *Dot1l-MM* and wildtype cells were plated in methylcellulose medium (StemCell Technologies) containing 10% ES serum (Hyclone), 5% protein-free hybridoma medium (Gibco-BRL), and cytokines SCF (100 ng/ml), LIF (1000 U/ml), EPO (2 U/mL), VEGF (5 ng/ml), IL-3 (5 ng/ml), IL-6 (5 ng/ml), IL-11 (5 ng/ml), GM-CSF (3 ng/ml), G-CSF (30 ng/ml), and M-CSF (5 ng/ml) (all cytokines were purchased from PeproTech). Colonies were analyzed for identity, size, and number after 7 days of *in vitro* culture.

### Analysis of Definitive Erythropoiesis in Yolk Sac

To assess definitive erythropoiesis, erythroid burst-forming unit (BFU-E), erythroid colony forming unit (CFU-E), and granulocyte and macrophage colony-forming unit (CFU-GM), assays were performed as described previously (Feng et al., 2010). Briefly, E10.5 yolk sac and embryos were collected by timed mating between *Dot1l-*MM heterozygous male and female mice. Embryos were used for determining the genotypes and yolk sacs were digested with collagenase to prepare single cell suspensions as described above. Approximately equal number of dissociated cells from *Dot1l-*MM and wildtype yolk sacs were plated in 35-mm culture dishes in M3434 methylcellulose medium (StemCell Technologies) containing cytokines SCF (100 ng/ml), EPO (2 U/ml), IL-3(5 ng/ml), IL-6 (5 ng/ml) (PeproTech) that promote definitive erythroid, myeloid, and mixed progenitor colony formation. The cells were cultured at 37°C for 10 days and scored according to the manufacturer’s recommendations. Single-colony area was measured with ImageJ software (National Institutes of Health).

### Inhibition of DOT1L Methyltransferase Activity in Mice

DOT1L methyltransferase activity was inhibited by administration of EPZ-5676 (Cayman Chemical). EPZ-5676 (35 μg/μl) was dissolved in ethanol and stored at −20°C. 4 weeks old mice were intraperitoneally injected with EPZ-5676 (35 μg/g of body weight, twice daily) diluted in 100 µl of 9:1 saline: ethanol solution as reported previously (Daigle et al., 2013). Control mice were injected with 100 µl of the 9:1 saline: ethanol solution. After 3 weeks of daily injections, mice were euthanized, and blood samples collected in presence of 3.8% sodium citrate solution (1/10th vol/vol). 10µl of blood was used for hemoglobin estimation using a Mission Plus Hemoglobinometer (San Diego, CA). 2.5 µl of each blood sample was smeared onto a glass slide to prepare a blood film following standard procedures. Blood films were air dried and stained with Hema three stains (Fisher HealthCare) following the manufacturer’s instructions. Stained blood films were imaged using brightfield microscopy. In addition to peripheral blood, bone marrows were collected from femurs and tibias of the hind limbs of each mouse. One half of the bone marrow cells were used for RNA and another half for protein extraction.

### Western Blot Analyses

Western blot analyses of H3K79 methylation was performed using whole body lysate of E8.5 embryos. Each of Dot1l-MM or wildtype embryos were lysed in 200 µl of 1XSDS sample buffer (Cell Signaling Technologies) and sonicated to shear genomic DNA. 20µl of the tissue lysate was heat denatured and electrophoresed on a 4–20% acrylamide gel (BioRad). Smaller proteins including histone H3 (∼17 KDa) were electrotransferred to a PVDF membrane using low amperage and short time (75 mAmp for 30 min), while high molecular weight proteins including DOT1L (∼185 kDa) were transferred with 100 mAmp for 6 h. Membranes were blocked with 5% skim milk in TBST and incubated with primary antibodies in blocking solution at 4°C overnight. A list of antibodies used this study is included in Supplemental Table S1. Levels of dimethyl H3K79 were detected by using a rabbit monoclonal antibody (Cell Signaling Technologies). Total H3 levels were detected as loading control using a mouse monoclonal antibody (Abcam). DOT1L was detected by using a rabbit monoclonal antibody (ABclonal) and alpha tubulin was detected by a mouse monoclonal antibody (Millipore) as loading control. After incubating with the primary antibodies, membranes were washed with TBST, blocked, and incubated with peroxidase conjugated anti-mouse, or anti-rabbit secondary antibodies (Jackson Immunoresearch, West Grove, PA) and the immunoreactivite signals were visualized with Luminata Crescendo HRP substrate (Millipore Sigma, Burlington, MA).

### RNA Extraction, and RT-qPCR Analyses

Total RNA was extracted using TRI Reagent (Sigma-Aldrich) according to manufacturer’s instructions. 1µg of total RNA from each sample was reverse transcribed by using High-Capacity cDNA Reverse Transcription Kits (Applied Biosystems, Foster City, CA). RT-qPCR amplification of cDNAs was carried out in a 10 µl reaction mixture containing Applied Biosystems Power SYBR Green PCR Master Mix (Thermo Fisher Scientific). Amplification and fluorescence detection of RT-qPCR were carried out on Applied Biosystems Quant Studio Flex seven Real Time PCR System (Thermo Fisher Scientific). The ΔΔCT method was used for relative quantification of target mRNA expression level normalized to Rn18s (18S rRNA). A list of qPCR primers is shown in Supplemental Table S2.

### Statistical Analyses

Each study group consisted of at least six individual mice. The experimental results were expressed as mean ± SE. Statistical comparisons between two means were determined with Student’s t-test while comparisons among multiple means were evaluated with ANOVA followed by Duncan post hoc test. *p*-values <0.05 were considered as significantly different. All statistical calculations were done with SPSS 22 (IBM, Armonk, NY).

## Results

### Generation of *Dot1l-MM* Mouse From Methyltransferase Mutant ES Cells

Methyl mutant E14 mES cells were generated by targeted replacement of the coding sequence of Asn 241 in *Dot1l* gene to Ala (Figure 1A) using CRISPR/Cas9 system and the mutant sequences were verified by Sanger sequencing (Figure 1B). Allele-specific PCR identified the desired monoallelic and biallelic mutant mES clones. We observed that the level of DOT1L expression in biallelic mutant mES cells were comparable to that of wildtype mES cells (Figure 1C). However, H3K79 methylation was absent, indicating loss of methyltransferase activity in *Dot1l-MM* mES cells (Figure 1D). The *Dot1l-MM* mouse was generated by microinjecting a sequence-verified monoallelic methyl mutant E14 mES clone into wildtype C57BL/6 blastocysts and transferring into pseudopregnant recipients. Chimeric mutant founder mice were identified by agouti spots in C57Bl/6 pups. The mutation in chimeras transferred successfully through the germline and heterozygous *Dot1l-MM* male and female mice were generated.

### Embryonic Lethality of *Dot1l-MM* Mouse Despite Normal Blood Formation

**FIGURE 2:**
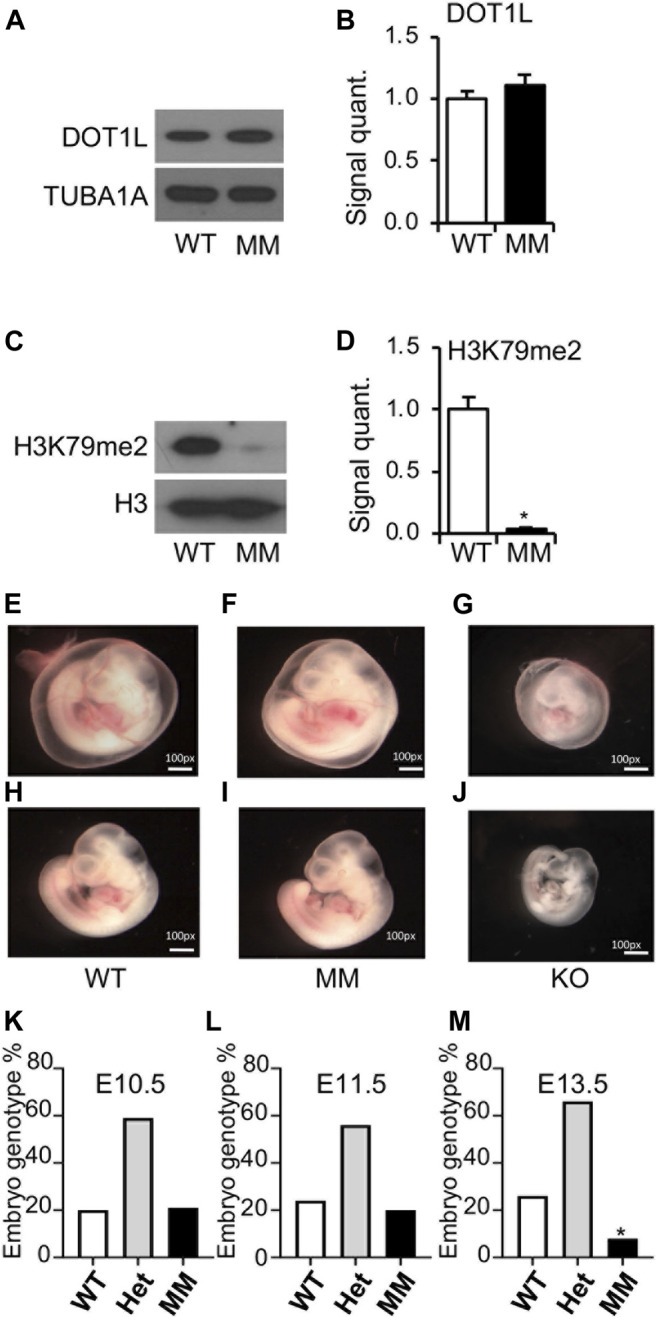
Embryonic lethality of Dot1l-MM embryos despite normal blood formation. E10.5 Dot1l-MM (MM) embryos possessed DOT1L protein expression comparable to that of wildtype (WT) embryos **(A,B)**. However, the level of di-methylated H3K79 (H3K79me2) was dramatically reduced in the Dot1l-MM embryos **(C,D)**. Representative pictures of E10.5 WT, Dot1l-MM, and Dot1l-KO embryos are shown in the image panels **(E–J)**. Dot1l-MM yolk sacs showed blood vessels remarkably higher in abundance than that of the Dot1l-KO yolk sacs, comparable to those of the WT **(E–G)**. Dot1l-MM embryos were also larger than Dot1l-KO and similar in size to WT embryos **(H–J)**. Despite their apparently normal size and comparable blood content, none of the embryos survived on E13.5 **(K,L,M)**. About 25% of the E13.5 Dot1l-MM embryos were still detectable by PCR genotyping, as the tissues had not yet been resorbed **(M)**. Data presented as mean ± SEM, n ≥ 6, and * indicates p ≤ 0.05.

E10.5 embryos were collected from timed mating of *Dot1l-MM* heterozygous males and females. Genomic DNA purified from the head of the embryos was used for genotyping, while the rest of the body was used for protein extraction and western blotting. Levels of DOT1L expression in the biallelic mutants were comparable to that of wildtype (Figure 2A), however, H3K79 di-methylation was barely detected in those *Dot1l-MM* embryos (Figure 2B). The *Dot1l-MM* embryos at E10.5 appeared to be similar in size compared to their wildtype littermates (Figure 2C). While *Dot1l-KO* embryos were much smaller and severely anemic, as previously reported (Feng et al., 2010) (Figure 2C). *Dot1l-MM* yolk sacs were well vascularized and contained blood (Figure 2C). Remarkably, blood in the *Dot1l*-MM embryos, which was visible in the heart and aorta-gonad-mesonephros (AGM) region, appeared similar to that of wildtype (Figure 2C). To determine embryonic lethality, embryos were also collected at E11.5, E12.5, E13.5 and genotyped. At E10.5, *Dot1l-MM* embryos showed a normal Mendelian ratio (Figure 2D). However, ∼10% of *Dot1l-MM* embryos were dead at E11.5 and none of the *Dot1l-MM* embryos were alive at E13.5 (Figure 2D).

### *Ex vivo* Primitive Hematopoiesis From *Dot1l-MM* Yolk Sac Cells

**FIGURE 3:**
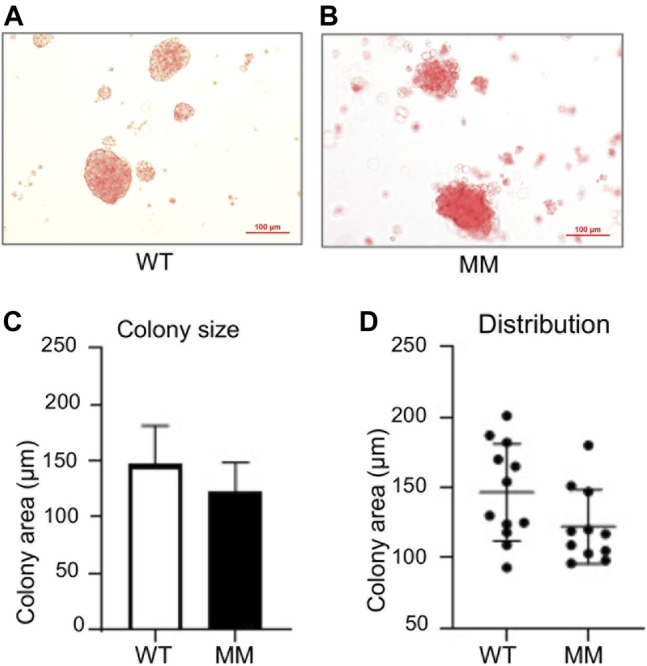
Primitive progenitors isolated from E8.5 Dot1l-MM yolk sacs form normal erythroid colonies. Wildtype (WT) and Dot1l-MM (MM) E8.5 yolk sac cells were cultured on methylcellulose medium containing cytokines that promote primitive erythroid lineage differentiation. Representative images of WT and Dot1l-MM erythroid colonies are shown in the upper panels **(A,B)**. Average colony area and size distribution is depicted in the lower panels **(C,D)**. We did not observe any remarkable differences in erythroid colony formation between Dot1l-MM and WT progenitor cells. Data presented as mean ± SEM, n ≥ 3.

The apparent similarity in blood formation between wildtype and *Dot1l-MM* yolk sac and embryos at E10.5 prompted us to examine primitive hematopoiesis in these embryos. To measure primitive hematopoiesis, *Dot1l-MM* and wildtype embryos and their yolk sacs were collected from E8.5 conceptuses after timed mating between *Dot1l-MM* heterozygous males and females. Genomic DNA extracted from the embryos were used for genotyping and yolk sacs were digested to collect the cells to establish primitive hematopoietic cultures. Approximately equal numbers of wildtype and *Dot1l-MM* yolk sac cells were plated on methylcellulose media for hematopoietic colony formation assay. In the colony formation assays, nucleated primitive erythrocytes from both *Dot1l-MM* or wildtype yolk sac cells formed distinct bright red colonies (Figures 3A,B). *Dot1l-MM* yolk sac cells formed primitive erythroid colonies of similar size, cellularity, and morphology as wildtype yolk sac cells (Figures 3C,D).

### *Ex vivo* Definitive Hematopoiesis From *Dot1l-MM* Yolk Sac Cells

**FIGURE 4:**
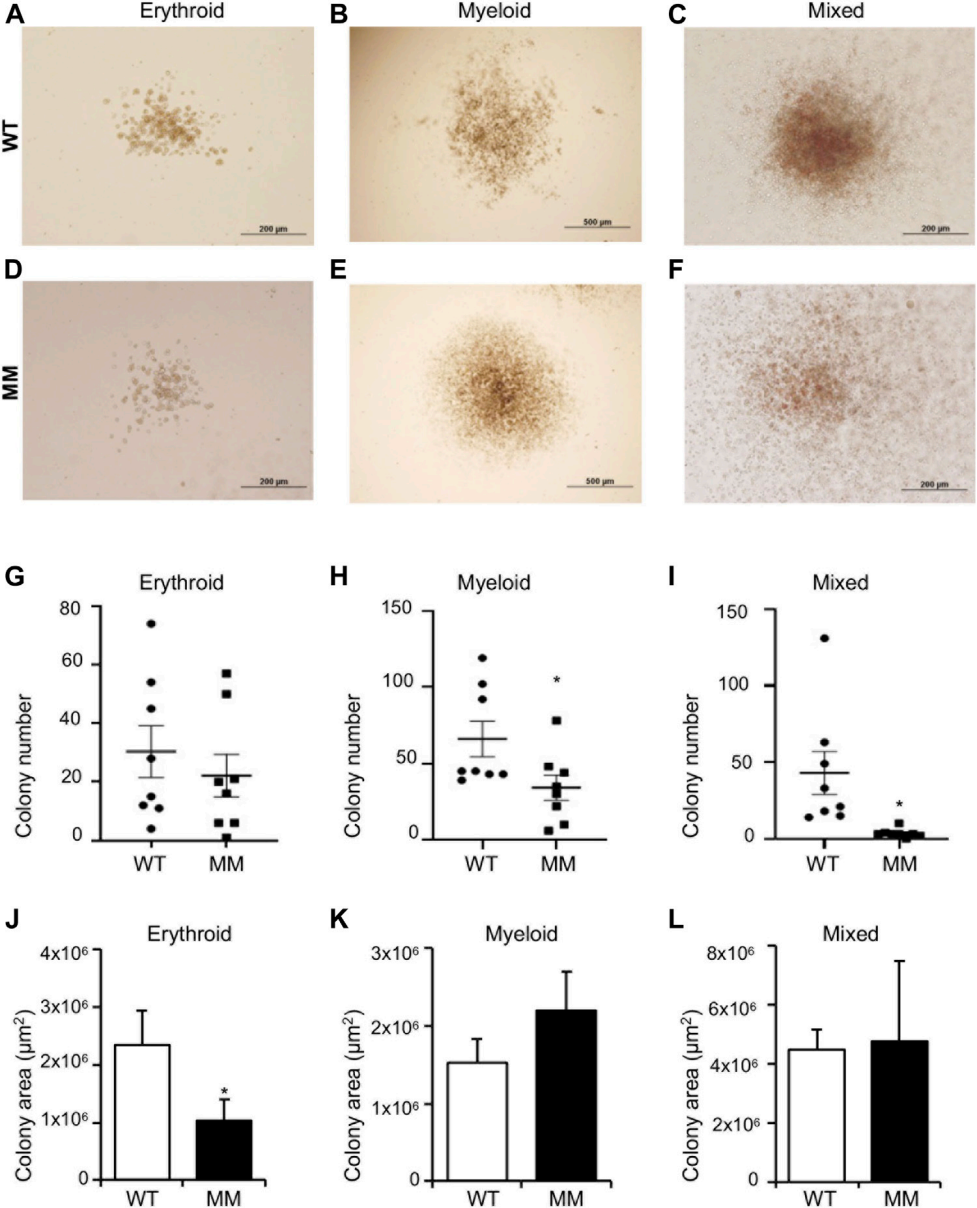
Dot1l-MM progenitors from E10.5 yolk sacs displayed altered erythroid, myeloid, and mixed colony formation. Equal numbers of wildtype (WT) and Dot1l-MM (MM) E10 yolk sac cells were cultured in methylcellulose medium containing cytokines that promote definitive erythroid, myeloid, and mixed hematopoietic lineage differentiation. Representative images of WT and Dot1l-MM erythroid (BFU-E) **(A,D)**, myeloid **(B,E)**, and mixed **(C,F)** colonies. The colonies were counted **(G–I)**, and the areas were measured **(J–L)** on day 10. Dot1l-MM progenitors formed similar numbers of erythroid colonies as WT, but they were relatively smaller **(G,J)**. Dot1l-MM progenitors formed significantly fewer myeloid **(H)** and mixed **(I)** colonies than those of the WT, but the few colonies that did form were similar in size as the WT colonies **(K,L)**. Data presented as mean ³ SEM, n ≥ 6, and * indicates p ≤ 0.05.

For definitive hematopoiesis assays, embryos and corresponding yolk sacs were collected on E10.5. DNAs extracted from the embryos were used for genotyping and similar number of cells isolated from the yolk sacs were plated on methylcellulose in presence of appropriate cytokines for colony formation. We observed that cells undergoing definitive hematopoiesis can differentiate into a much broader variety of cell lineages, including mature erythroid, and myeloid cells as well as multipotent cells and formed distinctive colonies. Cells from methyl mutant yolk sacs formed slightly fewer erythroid colonies than wildtype, and the difference was not significant (Figure 4A). In contrast, myeloid colony number was significantly reduced (Figure 4B) and the most striking result was the sharp (∼95%) reduction in mixed lineage colony formation (Figure 4). However, the average size of the methyl mutant erythroid, myeloid, or mixed colonies were of similar to that of wildtype (Figures 4D,F). Although the average areas and distribution of areas of the colonies were similar, the methyl mutant colonies had significantly less cellularity (Figures 4D–F) than the corresponding colonies formed by wildtype yolk sac cells (Figures 4A–C).

### Inhibition of DOT1L Methyltransferase in Young Mice

**FIGURE 5:**
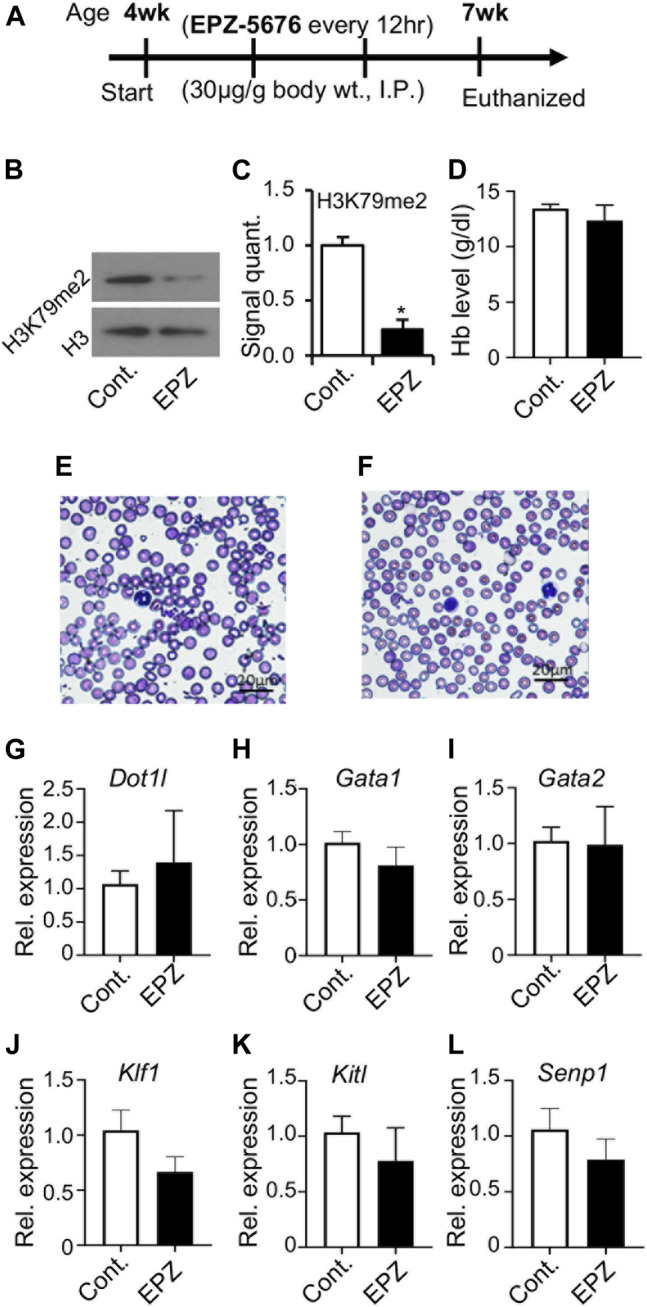
Inhibition of DOT1L methyltransferase activity with administration of EPZ-5676. Selective DOT1L methyltransferase inhibitor, EPZ-5676 (EPZ) was dissolved in 9:1, saline:ethanol and administered (30 μg/g body weight, intraperitoneally, twice daily) into 4-week old mice for 3 weeks **(A)**. Age-matched control mice (Cont.) mice were injected with 9:1 saline:ethanol for the same duration. After 3 weeks, of EPZ-5676 or vehicle-treated mice were euthanized, and blood and bone marrow were collected. EPZ treatment significantly reduced H3K79 di-methylation in bone marrow cells **(B,C)**. However, hemoglobin (Hb) levels were not decreased after EPZ treatment **(D)**, and a peripheral blood film showed apparently minimal-to-no changes in the blood **(E,F)**. RT-PCR analysis of expression of genes involved in blood development in bone marrow cells from Cont. or EPZ-treated mice showed no significant changes **(G–L)**. Data presented as mean ± SEM, n ≥ 6, and * indicates p ≦ 0.05.

We observed that the loss of DOT1L methyltransferase activity did not result in any significant effect on early embryonic erythropoiesis (Figures 2–4). So, we examined what affect, if any, the inhibition of DOT1L methyltransferase activity would have on peripheral blood. We injected a selective DOT1L methyltransferase inhibitor, EPZ-5676 into mice (35 μg/g of body wt, intraperitoneally, twice daily, for 3 weeks) (Figure 5A). At the end of this injection regimen, EPZ-5676 injected and vehicle treated (control) mice were sacrificed, peripheral blood and bone marrow were collected, and a number of parameters associated with erythropoiesis were assessed. We did not detect any significant differences in hemoglobin concentration between EPZ-5676 injected and control mice. The peripheral blood film also appeared similar between the control and EPZ injected mice (Figure 5F). We also did not observe any differences in DOT1L expression. However, H3K79 methylation in these cells was significantly diminished (Figure 5). Remarkably, we did not observe any significant differences in the expression of candidate genes involved in regulating erythropoiesis in bone marrow cells (Figure 3C).

## Discussion

We previously reported that DOT1L histone methyltransferase is essential for early embryonic hematopoiesis in mice (Feng et al., 2010). In *Dot1l-KO* mice, the mutation resulted in instability of *Dot1l* mRNA and loss of the protein (Feng et al., 2010). Loss of DOT1L protein led to lethal anemia in the knockout embryos (Feng et al., 2010). In the present study, we mutated a highly conserved Asn in the methyltransferase domain of DOT1L that can eliminate the ability of DOT1L to methylate H3K79 without altering its structure (Min et al., 2003). Using the *Dot1l-MM* animal model, we demonstrate that primitive erythroid development in the mouse yolk sac is independent of the histone methyltransferase activity of DOT1L. While primitive erythropoiesis occurred normally in the absence of DOT1L methyltransferase activity, it certainly affected some essential developmental processes, since the embryos failed to survive beyond E13.5, underscoring the importance of DOT1L methyltransferase activity during early embryonic development.

Yolk sacs of E10.5 *Dot1l-MM* embryos possessed prominent vascularization and blood, and embryos also showed the presence of red blood cells similar to that of wildtype (Figure 2). Since the *Dot1l-MM* embryos die between E11.5 and E13.5, we reasoned that the majority of hematopoiesis in these embryos occurred in the yolk sac. At E10.5, most of the circulating differentiated erythroid cells are derived from primitive hematopoiesis (McGrath et al., 2011). Anemia observed prior to E13.5 is attributable to impaired primitive erythropoiesis (Brotherton et al., 1979; Kingsley et al., 2004; McGrath et al., 2011; McGrath et al., 2015). Based on these prevailing concepts, we conclude that the process of primitive hematopoiesis remained intact in *Dot1l-MM* yolk sac. In sharp contrast, primitive erythropoiesis was substantially diminished in the *Dot1l-KO* yolk sac, and the embryos were severely anemic at E10.5 (Figure 2; (Feng et al., 2010). These observations suggest that a methyltransferase-independent role of DOT1L is essential for primitive erythropoiesis.

In contrast to primitive erythropoiesis, definitive hematopoiesis, *in vitro,* was impacted by the loss of methyltransferase function. In particular, the formation of *Dot1l-MM* myeloid and mixed colonies was significantly impaired (Figure 4). These observations suggest that the late stages of embryonic hematopoiesis require the intrinsic methyltransferase activity of DOT1L. Strikingly, formation of definitive erythroid colonies was less affected than the formation of either myeloid or mixed colonies. This is also in line with our observation following *in vivo* inhibition of DOT1L methyltransferase activity. Administration of EPZ-5676 into young mice markedly diminished the H3K79 methylation, without decreasing hemoglobin concentration even after 3 weeks of continuous treatment. Thus, erythroid progenitors seem independent of DOT1L methyltransferase activity. Further studies will be required to definitively identify and distinguish the DOT1L methyltransferase-dependent and -independent mechanisms during early embryonic and adult hematopoiesis.

**FIGURE 6:**
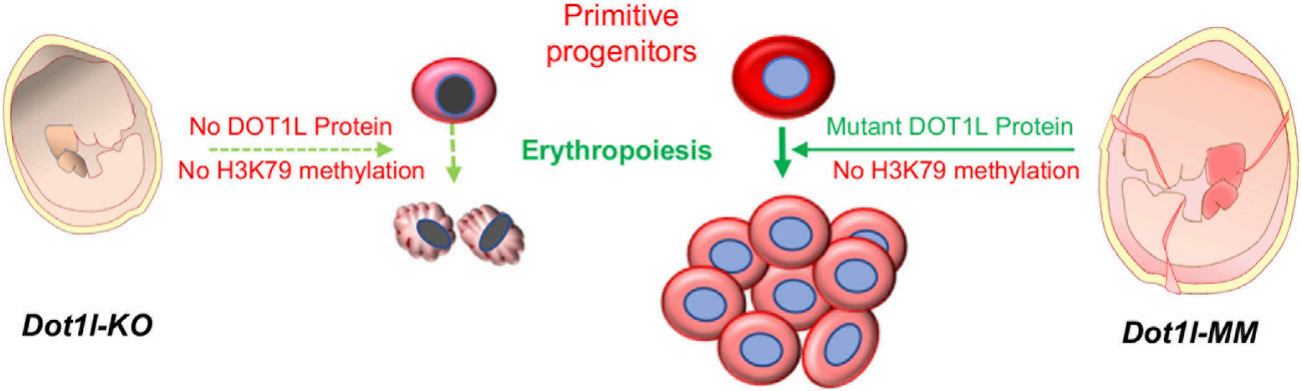
Primitive hematopoiesis in Dot1l-MM yolk sac. DOT1L is essential for early mammalian erythropoiesis. Absence of the DOT1L protein in the Dot1l-KO mouse leads to the lack of H3K79 methylation, which results in lethal anemia. A targeted point mutation (Asn241Ala) in the methyltransferase domain of DOT1L also causes the loss of H3K79 methylation in Dot1l-MM mice. However, the methyl mutant DOT1L protein is expressed at normal levels in these mice and allows primitive hematopoiesis to proceed. These findings suggest that a methyltransferase-independent mechanism of DOT1L is crucial for primitive erythropoiesis.

In both *Dot1l*-MM embryos and in bone marrow cells from mice treated with EPZ-5676, H3K79 methylation was substantially reduced (Figures 2, 5). However, in both cases it should be noted that a small amount of residual H3K79 methylation was detectable by Western blot analysis. It is formally possible that this residual activity may be sufficient to allow primitive hematopoiesis to proceed. However, we believe this is unlikely for two reasons: First, many other developmental processes are affected in the methyl mutant embryos, including definitive hematopoiesis. In addition, the methyl mutation results in embryonic lethality at about the same time as the knockout. Second, we have observed that about 40% of the differentially expressed genes (DEGs) in hematopoietic progenitor cells from the *Dot1l-*KO and *Dot1L-*MM yolk sacs are unique to either mutant group. These data indicate that very different transcriptional programs (some overlapping, some non-overlapping) are initiated in YS progenitors that either lack the entire DOT1L protein or lack the DOT1L intrinsic methyltransferase domain (i.e., DOT1L activity) (Borosha et al., 2020). The data are consistent with DOT1L possessing both methyltransferase domain-dependent and -independent functions in blood development (Schematized in Figure 6).

H3K79 methylation is the only recognized molecular function of DOT1L activity. DOT1L is essential for embryonic development and many vital cellular processes (Feng et al., 2010; Cattaneo et al., 2016; Pursani et al., 2018; Sutter et al., 2021). However, several recent studies have demonstrated evidence supporting methyltransferase-independent functions of Dot1p/DOT1L in yeast and embryonic stem cells, including nucleosome remodeling, histone exchange, H2B ubiquitination, transcriptional elongation, and cellular differentiation (Lee et al., 2018; van Welsem et al., 2018; Cao et al., 2020). The current study demonstrates for the first time an H3K79 methylation-independent role for DOT1L in a developmental process in a mammalian model system. Perhaps the most striking result of this study is the apparent difference in primitive erythropoiesis between the *Dot1l-KO* and the *Dot1l-MM*. Since the only defined activity associated with DOT1L is its role as a histone H3 methyltransferase, our data are consistent with the idea that there exist methyltransferase-independent pathways which are involved in hematopoiesis, particularly erythropoiesis. There may be many other important physiological functions of DOT1L that are independent of its H3K79 methyltransferase activity. Further implications of our results are far-ranging; DOT1L possesses activities that are potentially separable. If its essential regulatory roles can be separated from its roles in the pathophysiology of leukemogenesis and safely targeted, that will have overarching clinical implications.

## Data Availability

The raw data supporting the conclusion of this article will be made available by the authors, without undue reservation.
