# Supplementary material for: Primitive Erythropoiesis in the Mouse is Independent of DOT1L Methyltransferase Activity
Source: Front Cell Dev Biol. 2022 Jan 17;9:813503. doi: 10.3389/fcell.2021.813503 (PMC8802720; doi:10.3389/fcell.2021.813503)
Supplement: Supplementary file 1 [file Table1.docx]

**Supplementary Table 1. List of antibodies used in the western blot assays**

| **Target** | **Name of antibody** | **Manufacturer, Catalog number** | **Source, mono or polyclonal** | **Primary antibody dilution** | **Secondary antibody dilution** |
| --- | --- | --- | --- | --- | --- |
| H3K79me2 | Anti-H3K79me2 | Cell Signaling Technology, 5427 | Rabbit; monoclonal | 1: 4,000 | 1: 50,000 |
| H3 | Anti-H3 | Abcam, mAbcam 1220 | Mouse; monoclonal | 1: 4,000 | 1: 50,000 |
| DOT1L | Anti-DOT1L | Boster Bioscience, M00840 | Rabbit; monoclonal | 1: 2,000 | 1: 10,000 |
| TUBA1A | Anti-TUBA1A | Calbiochem, CP06 | Mouse; monoclonal | 1: 4,000 | 1: 50,000 |
