# Supplementary material for: Primitive Erythropoiesis in the Mouse is Independent of DOT1L Methyltransferase Activity
Source: Front Cell Dev Biol. 2022 Jan 17;9:813503. doi: 10.3389/fcell.2021.813503 (PMC8802720; doi:10.3389/fcell.2021.813503)
Supplement: Supplementary file 2 [file Table2.docx]

**Supplementary Table 2. List of primers used in the allele specific PCR and RT-qPCR assays**

| **Symbol** | **Reference seq** | **Forward primer** | **Reverse primer** | **Amplicon** |
| --- | --- | --- | --- | --- |
|  |  |  |  |  |
| *Dot1l* | NC_000076.7 | Wt-F-AATAACTTTGCCTTTGGTCCT | CmR-CTCCACAAGGGACAGCATGT | 426bp |
| *Dot1l-MM* | NC_000076.7 | Mt-F-GCTAATTTCGCTTTCGGACCA | CmR-CTCCACAAGGGACAGCATGT | 426bp |
| *Dot1l* | NM_199322.2 | 1189F-CATTGACCGCACCATACTTG | 1413R-GAGGGAGATGGCTTTTTGAC | 225bp |
| *Gata1* | NM_008089.2 | 758F-TGTGAGGCCAGAGAGTGTGT | 974R-TCCGCCAGAGTGTTGTAGTG | 217bp |
| *Gata2* | NM_008090.5 | 1099F-CCCTAAGCAGAGAAGCAAGG | 1220R-CACAGGCATTGCACAGGTAG | 122bp |
| *Klf1* | NM_010635.3 | 892F-GCGGGAAGAGCTACACCAAG | 1051R-AAGGGACGATGTCCAGTGTG | 160bp |
| *Kitl* | NM_013598.3 | 829F-ACTCGGGCCTACAATGGAC | 1059R-CCACTGTGCGAAGGTAACAA | 231bp |
| *Senp1* | NM_001379573.1 | 1051F-TGCAAATGGACAATGGAGAA | 1236R-GCAGGCTTAATGGGAAATGA | 186bp |
| *Rn18s* | NR_003278.3 | 1617F-GCGATTATTCCCCATGAACG | 1740R-GGCCTCACTAAACCATCCAA | 124bp |
